# Etiology and Treatment of Peri-Implant Soft Tissue Dehiscences: A Narrative Review

**DOI:** 10.3390/dj10050086

**Published:** 2022-05-16

**Authors:** Charalampos Kaddas, Eirini Papamanoli, Yiorgos A. Bobetsis

**Affiliations:** 1 Private Practice, 115 27 Athens, Greece; e.papamanoli@gmail.com; 2 Department of Periodontology, School of Dentistry, National and Kapodistrian University of Athens, 115 27 Athens, Greece

**Keywords:** buccal peri-implant soft tissue dehiscence, classification, connective tissue, dental implants, peri-implant recession, soft tissue dehiscence, surgical coverage

## Abstract

Implant soft tissue dehiscences compromise not only the aesthetics of the supported restorations but implant survival in the long run. The aim of this narrative review was to briefly present the causative factors of buccal peri-implant soft tissue dehiscences (PSTDs), how these are classified, and the current therapeutic approaches. Implant malposition and the thin peri-implant phenotype are the two major determinants for the occurrence of PSTDs, but other risk factors have also been identified. The most common surgical procedure for treating PSTDs is the split-thickness coronally advanced flap combined with either a connective tissue graft or acellular dermal matrix materials. However, depending on the class and subtype of the dehiscence, the combination of surgical techniques with modifications in the restoration may further ameliorate the final result. In general, within a five-year follow-up period, most techniques lead to a satisfactory aesthetic result, although full coverage of the implant/abutment surface is not always achievable, especially in more extended lesions.

## 1. Introduction

Dental implants have been proven to be a reliable solution for the successful rehabilitation of missing or nonrestorable teeth with favorable long-term survival rates [1,2]. However, today, implant survival is not the sole objective of implant dentistry. Optimal aesthetics and high patient satisfaction are considered the game changers in clinical practice, and therefore, the need for measurable outcomes/parameters is obvious [3,4]. For this reason, different indices, such as the pink and the pink/white aesthetic scores, have been proposed to help clinicians evaluate, in a more objective manner, the aesthetics of the peri-implant soft tissues and of the implant-supported prostheses. The facial contour and the level of the soft tissue margin are significant variables of these indices [5,6,7]. Recently, Zucchelli et al. introduced a new index (IDES) specifically for PSTD, in which the evaluation begins at least six months after the surgical attempts for coverage [8]. Specifically, four parameters are evaluated, which are the level of the soft tissue margin (STM), the peri-implant papillae height (PPH) compared with the homologous tooth, the peri-implant mucosa color, and the peri-implant mucosa appearance (PMA). The highest score that can be acquired is 10, and after examining a total of 51 cases with four different evaluators, the authors concluded that there was a high degree of agreement among different clinicians and among different time points of evaluation by the same clinician. Therefore, the index constituted an objective and reproducible means of evaluation of PSTD cases. Furthermore, the subjective perception of aesthetics by the patient is an important parameter of patient-reported outcomes measures [4,7]. Thus, a greyish hue visible through the mucosa, exposure of the abutment or of the implant surface, and discrepancies on the soft tissue contour due to buccal mucosa recession in the aesthetic zone are all factors that usually lead patients to become dissatisfied with their treatment outcome [9,10].

In 2017, in the World Workshop of EFP and AAP, new case definitions were proposed for peri-implant disease [11]. More specifically, peri-implant mucositis was defined as a plaque-induced inflammation of the peri-implant mucosa where bleeding and other clinical signs of inflammation are present. On the other hand, periimplantitis was defined as a plaque-associated pathological condition characterized by inflammation in the peri-implant mucosa and subsequent progressive loss of the supporting bone. These entities, regardless of their high prevalence, are not the only conditions that affect the success and survival of dental implants. Buccal peri-implant soft tissue dehiscences (PSTDs) develop by the apical shift of the facial mucosal margin of the implant-supported prostheses. Although different reference points have been used, the gingival margin of the adjacent/contralateral natural teeth has been suggested to be more reliable for recession evaluation [12]. Recently, Zucchelli et al. (2019) classified the PSTDs of single implants, not diagnosed with peri-implantitis, and proposed the most suitable treatment modality for each class [13]. According to this classification, Class I comprises cases where the soft tissues are at the correct level compared with the adjacent teeth, but the color of the implant or abutment reflects through the soft tissues, most likely because of the inadequate thickness of keratinized tissues. Class II involves cases where the soft tissue margin of the implant restoration is more apically positioned compared with the homologous natural tooth, while also the implant-supported crown is located palatally to the “imaginary curve” formed by the profile of the natural teeth at the level of the soft tissue margin. Lastly, the third and fourth classes include cases where the soft tissue margin is placed more apically, while the crown profile is located facially to the “imaginary curve” and the implant head is positioned more palatally (Class III) or facially (Class IV) to the straight imaginary line that connects the profile of the adjacent teeth. In addition, Classes II and III can be further divided into three distinguished subtypes. Subtype a refers to cases where both (mesial and distal) papillae tips are located 3 mm coronally to the ideal position of the soft tissue margin, whereas subtype b regards cases where at least one papillae tip is located less than 3 mm coronally to the ideal margin. Lastly, subtype c is identified as at least one papilla being located at the same level or more apically compared with the ideal height of the soft tissue margin of the implant-supported prosthesis. Barootchi et al. proceeded to assess the reliability of the aforementioned classification [14]. The investigators chose 12 general practitioners and 10 periodontologists to participate in the study, each of whom evaluated 25 single PSTD cases with the proposed scheme. The statistical analysis of the retrieved results showed that this classification system provided reproducible evaluations, both among examiners of different backgrounds and among examiners of the same specialization and skill. The only slight difference was that the gold-standard examiner’s evaluation was more in sync with the assessment results of dentists trained in periodontology.

Peri-implant soft tissue dehiscence has been observed after the prosthetic rehabilitation of dental implants and seems to occur mainly after the first 6 months of implant loading and may continue to increase with time [15,16]. In a recent cross-sectional study by Romandini et al., 272 anteriorly placed implants with at least one year of loading were examined for PSTDs in a total of 92 patients [17]. Buccal soft tissue deficiency was found in 12% of implants without periimplantitis, and only in 0.6% of them was the deficiency characterized as severe (>2 mm). However, this particular study used a different reference point than the definition proposed by Zucchelli et al. in 2019 [13]. Specifically, Romandini et al. used the Sanz-Martin et al. definition, where PSTD depth is measured if exposure of the implant surface, implant neck, or abutment is present, which is irrelevant to the height of the existing implant-supported crown [18]. On the contrary, Tavelli et al. used the Zucchelli et al. definition and classification to assess 153 cases that included 176 implants. The prevalence rates were 54.2% at patient level and 56.8% at implant level [19]. Moreover, they underlined that in cases where the prosthesis had a greater height than the homologous tooth or the abutment/implant fixture was visible were the most commonly found. Garabetyan et al., in a retrospective study, assessed the alterations of peri-implant soft tissues in 90 implants placed at different times postextraction, and almost half of them presented a combination of guided bone regeneration (GBR) and connective tissue graft (CTG) [20]. A 5.6% incidence of midbuccal mucosa recession was found after a mean 4.5-year follow-up of implant loading, while at 1, 3, and 5 years, the percentage of recession-free implants was 98.9%, 97%, and 89.6%, respectively. On the contrary, in their review, Chen and Buser claimed that PSTDs following immediate implant placement should be considered as a common finding ranging from 9 to 45% of the sites, while the overall mean frequency of the sites with >1 mm of buccal mucosa recession was 21% [21]. This rate seemed to be lower for implants with early placement protocol. 

The aim of this narrative review was to present the main factors associated with greater risk for the development of PSTDs and describe the main surgical techniques for the coverage of these deficiencies.

## 2. Risk Factors for PSTD

Different parameters such as implant position, peri-implant biotype, width and thickness of keratinized mucosa, peri-implant bone crest height and thickness, implant design, and surgical implant placement protocol have been evaluated as possible risk factors for the development of PSTDs. Τhe degree to which some of these parameters affect the peri-implant soft tissue stability remains controversial in the literature [16,18,20,22,23,24,25,26,27,28,29,30].

A major determinant for the occurrence of PSTD has been proven to be the buccolingual malposition of the implants [17,18,22,24]. Implants with a buccal position of the implant shoulder relative to the line drawn between the cervical margins of the adjacent teeth had three times more recession compared with implants of which the shoulder was placed lingually to this line (1.8 mm and 0.6 mm, respectively) [22]. For this reason, Buser et al. recommended that the implant shoulder should be placed 1–2 mm palatal to the line of emergence of the adjacent teeth [25]. In a cross-sectional observational study, implants positioned >1 mm outside the alveolar envelope, as determined by cone beam computed tomography (CBCT), were 34 times more likely to present PSTD (odds ratio (OR) 34.65, *p* < 0.001) [18]. The association between the buccally located implant shoulder and the midfacial recession was also demonstrated in a retrospective cohort study by Cosyn et al. with an OR 17.2 [24]. Similarly, Romandini et al. identified excessive buccal implant position as a risk factor for buccal peri-implant soft tissue dehiscence [17]. On the contrary, in another cross-sectional study, the implant position was not found to significantly affect the facial marginal mucosal level [23]. In this study, the authors claimed that the implant fixture angle was a considerable factor that increased the risk for PSTD in an inverse way. Thus, implants with more proclined fixture position are expected to demonstrate a more apical displacement of the facial marginal mucosal level.

The thin peri-implant biotype has also been identified as a significant risk factor for the presence of PSTD [16,17,20,22,23]. Specifically, peri-implant sites with thin biotype exhibited significantly greater risk for development of PSTD than sites with thick biotype (OR 18.8, *p* = 0.01) [23]. In a prospective study, Kan et al. reported significant differences in the mean facial gingival changes between sites with thick and thin biotype at 1 year post-implant placement (−0.25 mm vs. −0.75 mm, respectively). This difference was further increased when implants were assessed at the most recent follow-up (−0.56 mm vs. −1.50 mm, respectively) [16]. In their study Evans and Chen observed a higher frequency of recession of >1 mm after 19 months of immediate implant placement in sites with thin rather than thick biotype [22]. Therefore, sites with thin tissue biotype should be considered as more prone to PSTD, especially when implants are also placed in a buccal position [17,22].

The clinical significance of the presence of an adequate band of keratinized mucosa around dental implants has also been widely investigated in the literature, with controversial results [31,32]. The protective effect of a keratinized mucosal zone of >2 mm against the occurrence of PSTD was reported by Sanz et al. [18]. In addition, Roccuzzo et al. observed significantly less soft tissue recession for implants surrounded by keratinized mucosa than those with alveolar mucosa after a 10-year follow up (0.16 ± 0.39 mm vs. 2.08 ± 0.71 mm, *p* = 0.0001) [30]. Tavelli et al. also recently found that both inadequacy of mucosal thickness and reduced keratinized mucosa width were significantly correlated with PTSD occurrence [19]. These results were in concordance with the findings of a meta-analysis wherein the inadequate width of keratinized mucosa was correlated with higher values of mucosa recession and loss of attachment [33]. 

The effect of the height and thickness of the buccal bone on the development of PSTDs appears to be ambiguous in the available studies. In a CBCT study, Benic et al. observed that implants with absence of the buccal bone at 7 years after immediate placement demonstrated 1 mm more apical displacement of the marginal mucosa level than implants with intact buccal bone [26]. Nisapakultorn et al. noted also that the facial crest level significantly affected the buccal marginal mucosa level, while the facial crest thickness did not seem to have a significant effect [23]. Tavelli et al. also identified increased buccal bone distance (BBD) as a significantly associated parameter to PTSD development [19]. However, contradictory findings came from another CBCT study, in which the buccal bone thickness was found to have a significant negative correlation to both buccal vertical bone loss and soft tissue recession. For this reason, the authors suggested that a minimum labial bone thickness of 2 mm should be present at the time of implant placement with both immediate and two-stage protocol in order to maintain long-term tissue stability [29].

In an RCT study, 28 implants with an osseous dehiscence defect (≤5 mm) at implant placement were randomized to a guided bone regeneration (GBR) or a spontaneous healing (SH) group. Despite the fact that the SH group demonstrated significantly greater vertical buccal bone loss at the re-entry surgery 6 months after implant placement and at 18 months after implant loading, there were no significant differences at the marginal mucosa level between the two groups after 18 months [28]. This was in agreement with the findings of two clinical studies in which buccal bone dehiscence was presented without a concomitant recession of the buccal mucosa level [18,34]. 

The level of implant positioning at the apicocoronal dimension could also have a significant effect on the buccal soft tissue level [23,25]. Nisapakultorn et al. reported that increased distance between the contact point and the implant platform increased the risk of buccal marginal mucosa recession with an OR of 2.3 (*p* = 0.005) [23]. Moreover, the greater distance between the contact point and the bone-to-implant level, as well as the interproximal crest level, was, the higher the risk for PSTD was. The correlation between midfacial mucosa recession and papilla level was found to be irrelevant [23]. Nevertheless, in a study by Garabetyan et al., the authors claimed that alterations in papilla height could also induce changes at the buccal marginal mucosa level [20]. 

The immediate implant placement protocol has also been associated with midfacial recession [7,21,29,35,36,37,38], especially in the presence of risk indicators such as thin tissue biotype, facial implant malposition, and thin buccal bone wall [21,36,38]. In a systematic review, immediate implant placement demonstrated a higher median frequency of recession of >1 mm of the midfacial mucosa (range 9–41%, median 26% of the sites) in comparison with early implant placement after soft tissue and partial bone healing (no sites with recession > 1 mm) [36]. On the other hand, in a case series study, advanced midfacial recession (>1 mm) was found only in 7% of immediate implants, while the respective percentage for conventional installed implants was 43%. This difference could be attributed to the flapless procedure that was performed in 9 of the 16 immediately placed implants, which noted a significantly lower midfacial recession of 0.89 mm at 52 weeks compared with the flap approach [39]. In addition, immediate provisionalization enabled the preservation of the soft tissue contour after tooth extraction and immediate implant placement [35]. In an RCT, De Rouck et al. observed a 2.5- to 3-fold larger amount of midfacial recession for immediate single tooth implants with delayed restoration (provisionalization 3 months after implant placement) than those with immediate restoration [40]. The mean difference between the two groups at 12 months was 0.75 mm and was statistically significant (1.16 mm for delayed vs. 0.41 mm for immediate restoration group, *p* = 0.005). On the contrary, in another RCT, in which implants were placed at healed sites, there were no differences in the midfacial soft tissue level between immediate and conventional restoration groups [41].

With regard to the implant design, a recent case-control study associated one-piece implants with higher risk for PSTD [18]. However, in a prospective randomized controlled clinical study, the authors did not find differences at the peri-implant soft tissues between one- and two-piece implants 1 year after loading [27]. Buser et al. noted the significance of the proper selection of implant platform and neck in relation to the mesiodistal and buccolingual dimensions in order to achieve better aesthetic outcomes [25]. In an RCT study, immediately placed implants restored with the platform switching technique demonstrated significantly more favorable results in terms of midbuccal mucosa recession in comparison with implants restored with platform matching technique 2 years after loading [42]. In contrast to these findings, Zuiderveld et al. claimed that the platform switching technique was not a determinant for the midbuccal mucosa level [43]. Interestingly, the time of function of an osseointegrated and loaded implant and the presence of an adjacent implant are two factors that were also recently found to be associated with buccal soft-tissue dehiscence in a statistically significant manner [19]. 

Finally, behavioral factors such as smoking have also been associated to soft-tissue dehiscence around implants. Indeed, Raes et al. demonstrated the impact of smoking on midbuccal recessions on 95 patients, concluding that smoking contributed to greater recession [44].

## 3. Surgical Techniques for the Treatment of PSTD

The majority of surgical approaches for the treatment of PSTD combine a coronally advanced flap (CAF), with or without vertical incisions, with a CTG or acellular dermal matrix (ADM) or collagen matrix (CM) [9,10,45,46,47,48,49,50,51,52]. Other types of techniques have also been performed, but mainly in case reports [53,54,55,56] (Table 1).

Zucchelli et al. proposed the most appropriate treatment protocol for each of the four classes of dehiscence according to their classification [13]. Thus, in cases wherein the height of both papillae is ≥3 mm (subclass a), the coronal advancement and suturing of the flap with CTG is sufficient for the treatment of Class I and II. However, a combined prosthetic–surgical approach [10] with or without abutment replacement was recommended for Class IV and III, respectively, to augment the interproximal soft tissues and increase the vascular supply to the flap. In cases wherein the height of at least one papilla is <3 mm, a prosthetic–surgical approach was proposed for all Classes except Class IV, in which soft tissue augmentation with submerged healing was required. Soft tissue augmentation with submerged healing was also recommended to be performed in Classes II and III with concomitant absence of at least one papilla; on this occasion, as in Class IV, the implant should be removed.

The surgical–prosthetic approach was described by Zucchelli et al. in 2013 [10]. According to this approach, the implant prosthetic crown is removed at least one month prior to surgery, and the abutment is reduced and polished in order for a new finishing line to be created. Consequently, provisional restorative crowns are placed, allowing proper postsurgical soft tissue healing. After the mechanical treatment of the exposed implant surface with diamond burs, the dehiscence is treated with a CAF and CTG, the latter of which derives from the de-epithelization of a free gingival graft harvested from the palate. A 1-year follow-up after final restoration revealed complete dehiscence coverage in 75% of the cases and significant improvement in patients’ aesthetics in terms of VAS and PES/WES scores. At the 5-year follow-up, the percentage of complete coverage as well as patients’ aesthetics scores remained stable. In addition, the soft tissue thickness and keratinized tissue height increased significantly both at 1- and 5-year follow-up compared with baseline. Also very important was the absence of peri-implant mucositis, which was attributed to the brushing technique and maintenance care program [10,48]. The successful outcome of this technique derived from the augmented interdental tissues, which provided greater interdental beds for the graft and for the surgical papillae [10,48]. However, the surgical–prosthetic technique increases the time and cost of treatment. 

In another prospective study by Burkhardt et al., 10 patients were treated for soft tissue dehiscence with CAF and CTG [45]. In the 6-month postoperative evaluation, the mean dehiscence coverage was 66%. Despite the satisfactory degree of coverage, the authors concluded that total coverage around implants could rarely be achieved. It is possible that the inferior coverage outcomes reported by Burkhardt et al. could be attributed to the use of deep palate CTG, which is richer in fatty and granular tissue than de-epithelialized free gingival graft, which contains denser collagen fibers and is consequently more stable and less prone to contraction [57,58]. 

In an RCT, Anderson et al. compared the clinical outcomes of CAF with those of CTG and ADM for the treatment of PSTDs in a group of seven (control group) and six patients (test group) [46]. After an observational period of 6 months, the control group demonstrated 40% recession coverage, while the respective percentage for the test group was 23%. Although the differences in terms of clinical and aesthetic outcomes were not statistically significant between the two groups, the control group presented a more uneventful healing. The authors concluded that it was the underlying bone morphology and not the soft tissue biotype that determined the soft tissue treatment outcomes. Partial coverage of a baseline 3 mm recession was also reported in a case report study in which a triangular-shaped incision was performed for a CAF combined with ADM [49]. 

In a prospective study of Roccuzzo et al., 16 patients were treated for single implant dehiscence with a split-thickness envelope flap and CTG derived from the maxillary tuberosity [9]. This technique was proposed to be performed for the treatment of shallow dehiscence (2.0 ± 0.7 mm) with intact interproximal tissue, as the authors avoided the use of vertical incisions for greater coronal flap movement. The mean reduction in the dehiscence at the 1-year follow-up was 1.7 mm (range 1.4–2.0 mm), which was statistically significant. Complete coverage was achieved in 56.3% of the cases. The favorable results remained stable for the 13 patients who attended the 5-year follow-up, while complete coverage was still present at 62% of the implants [47].

An envelope (pouch) technique in conjunction with a subepithelial connective tissue graft (SCTG) was also used for the treatment of two peri-implant midfacial recessions of 1.5 and 2 mm. Both cases were left with a residual dehiscence of 0.5 mm after both 3 and 9 months [51]. In another prospective study, Schallhorn et al. performed a pouch flap with collagen matrix at 35 implant sites with soft tissue deficiencies [50]. Although the soft tissue thickness and the KT height were increased significantly at a 6-month follow-up, the authors did not observe any significant change in the mean recession.

Frisch et al., in a retrospective case series study, evaluated the clinical outcomes of a new surgical approach using partially epithelialized connective tissue grafts (PECTGs) [52]. The aim of this technique was to augment the KT height and thickness, as well as to cover peri-implant soft tissue recessions with a mean depth of 2.4 mm. The authors prepared a split thickness flap, and a PECTG was harvested from the palate. Subsequently, the graft was sutured with the KT portion towards the local KT tissue, and the connective tissue portion was covered by the mucosal flap. At a mean 5-year follow-up, all 22 implants demonstrated significant recession coverage of 88%, while complete coverage was observed in 64% of them. In addition, significant gains in terms of KM width and thickness were achieved. The PECTG technique was proven to be effective for the treatment of PSTDs, although a larger sample size is needed for more solid conclusions.

Other surgical [53], prosthetic [54], and combined surgical–prosthetic techniques [55,56] have also been described for the management of PSTD, mainly in case report studies. Lee et al. suggested an envelope technique with a modified vestibular-incision tunnel approach (VISTA technique) and a connective tissue graft [56]. The authors found very satisfactory results with regard to tissue height and width. Ueno et al. used a semilunar coronally positioned flap with an SCTG for the treatment of a PSTD around two posterior implants in the maxilla [53]. They reported successful coverage of the PSTD, which was maintained after 9 months. In a recent case report, Yang et al. corrected the compromised aesthetics of an anterior buccally malpositioned implant with midfacial recession [55]. For this purpose, they applied digital prosthodontics using the one-step zirconia coping technique and a tunneling surgical technique with a SCTG. The gingival margin was repositioned 2.9 mm coronally, and only a 0.5 mm recession was observed 3 years later. In their case, Schoenbaum et al. replaced the initial abutment and implant crown with a new provisional screw-retained composite resin and titanium interim restoration with an undercontoured emergence profile [54]. After 3 months, the midfacial mucosa migrated 1 mm coronally, and the final restoration was placed.

Taking into account all the aforementioned studies, it is clear that the treatment of mucosal recessions around dental implants requires advanced surgical skills and does not always guarantee the complete coverage of implant dehiscence. Moreover, the results presented should be examined with caution, since several limitations follow these studies. In particular, all investigations addressed shallow recessions, up to 3 mm [45,46,47,48,52]. In addition, among studies, different reference points to evaluate the peri-implant recession, as well as various indices for the assessment of the aesthetic outcome, have been used. Therefore, direct comparison of the clinical results is not always easy or meaningful [8,12]. In order to overcome this problem in future studies, Zucchelli et al. introduced a new Implant soft tissue Dehiscence coverage Esthetic Score (IDES) for a more objective evaluation of the treatment outcome that incorporates and standardizes both clinical and aesthetics outcomes, as mentioned above [8]. 

Another point worth discussing is the timing of the intervention. Since these surgical procedures are relatively unpredictable, it is reasonable to advocate the notion that prevention is always better than treatment. Therefore, it has been supported that it is preferable to perform soft tissue augmentation procedures before or at the time of implant placement or at the second stage surgery (re-entry) rather than after implant loading [59,60]. This is even more critical in immediate implant placement, since the dimensional changes of the alveolar ridge after tooth extraction are not prevented [61] and result in a higher incidence of midfacial recession [21]. Therefore, especially in cases with thin biotype or buccal bone dehiscence, immediate implant placement should be performed with concomitant bone augmentation and soft-tissue thickening in order to compensate for bone remodeling and the subsequent soft tissue contraction after tooth extraction [35,62]. This is in accordance with the findings of a meta-analysis wherein CTG with immediate implant placement had a significant protective effect against the occurrence of midfacial recession [63].

The long-term stability of peri-implant soft tissues after surgical intervention has been assessed by few studies with a mean follow-up of 5 years [47,48,52]. At this time, point soft tissue parameters has remained stable with minor nonsignificant changes. The maintenance care phase is always a very important aspect to be taken into consideration by clinicians. Modern, minimally invasive means of implant cleaning, such as air-abrasive decontamination with erythritol powder, damage neither the implant surface nor the soft tissues and remove all bacterial biofilms, contributing to the long-term stability of implant restorations [64].

In conclusion, buccal peri-implant soft tissue deficiencies developed after implant loading could compromise patients’ aesthetics. After immediate implant placement, the possibility for PSTD development is increased, especially when other risk factors such as implant malposition and thin biotype coexist. The most studied surgical technique for the treatment of these deficiencies is the coronally advanced flap with a CTG. However, complete implant coverage is not always feasible. The only available RCT, by Anderson et al., included a rather small sample size, while the assessment of the outcome was made within the first six months after the operation [46]. Therefore, there is a lack of randomized controlled trials that evaluate the effectiveness of different surgical approaches and biomaterials used. More studies with larger sample sizes and comparable outcome measures are necessary to evaluate other surgical approaches.

## Figures and Tables

**Table 1:** Surgical techniques for the treatment of PSTD and clinical outcomes.

Authors (Year)	Study Design	Number of Implants	Follow-Up	Surgical Technique	Soft Tissue Parameters Examined	Results
Burkhardt et al. (2008) [45]	Prospective study	10	6 m	CAF + CTG	STD coverage, PPD, KM width	Mean STD coverage: 66 ± 18% ΔPPD: 0.2 ± 1.28 mm KM width: −0.2 ± 1.1 mm
Mareque-Bueno S. (2011) [49]	Case report	1	6 m	CAF + ADM	STD coverage, PPD, KM width	Partial coverage of a 3.0 mm STD PPD remained stable (2 mm) ΔKM width: 1 mm
Cosyn et al. (2012) [51]	Prospective study	2	3 and 9 m	Envelope (pouch) technique + CTG	STD coverage	Initial STD depth 1.5 and 2.0 mm Residual STD depth at 3 and 9 m: 0.5 mm for both cases
Zucchelli et al. (2013) [10]	Prospective study	20	12 m	Surgical–prosthetic approach (abutment modification and CAF + CTG)	STD coverage, CAL gain, PPD, KM width, STT	ΔSTD depth: −2.62 ± 0.81 mm Mean STD coverage: 96.3% Complete coverage: 75% of cases CAL gain: 3.02 ± 1.06 mm ΔPPD: 0.40 ± 0.73 mm ΔKM width: 0.57 ± 0.41 mm ΔSTT: 1.58 ± 0.21 mm
Anderson et al. (2014) [46]	Randomized controlled trial	Control group: 7 Test group: 6	6 m	Control group: CAF + SCTG Test group: CAF + ADM	STD coverage STT, Soft tissue concavity dimensions	STD coverage Control group: 40% Test group: 23% STT increased by Control group: 63% Test group: 105% Concavity correction Control group: 82% Test group: 96%
Roccuzzo et al. (2014) [9]	Prospective study	16	12 m	Envelope (pouch) flap + CTG	STD coverage, PPD	ΔSTD depth: −1.7 ± 0.7 mm Complete coverage at 56.3% of the cases ΔPPD: 0.4 ± 0.64 mm
Lee et al. (2015) [56]	Case report	1	12 m	modified VISTA technique (envelope-tunnel flap) + CTG	Soft tissue width and height	Both soft tissue width and height increased
Schallhorn et al. (2015) [50]	Prospective study	N/A	6 m	Pouch flap + collagen matrix	STD coverage, PPD, STT, KM width	ΔSTD: −0.1 ± 0.7 mm ΔPPD: −0.5 ± 1.0 mm ΔSTT: 0.7 ± 0.8 mm ΔKM width: 0.7 ± 1.2 mm
Ueno et al. (2015) [53]	Case report	2	9 m	Semilunar coronary positioned flap + SCTG	STD coverage, STT, KM width	Full STD coverage at both implants ΔSTT: 2 mm for #15, 3 mm for #16 ΔKM width: 2 mm for both implants
Roccuzzo et al. (2018) [47]	Prospective study	13	5 y	Envelope (pouch) flap + CTG	STD coverage, PPD	ΔSTD depth: −1.7 ± 0.7 mm Mean STD coverage: 86 ± 19% Complete coverage in 62% of the cases ΔPPD: 0.2 ± 0.72 mm
Schoenbaum et al. (2018) [54]	Case report	1	5 m	Prosthetic approach	STD coverage	Complete coverage of an initial 1 mm STD
Zucchelli et al. (2018) [48]	Prospective study	19	5 y	Surgical–prosthetic approach (abutment modification and CAF + CTG)	STD coverage, CAL gain, PPD, KM width, STT	Mean STD coverage: 99.2% Complete coverage: 79% of the cases CAL gain: 3.0 mm (2.5–3.5 mm) PPD remained stable ΔKM width: 1.0 mm (0.5–2.0 mm) ΔSTT: 1.8 mm (1.6–2.1 mm)
Frisch et al. (2020) [52]	Case series	22	5 y (mean follow-up)	Repositioned flap + PECTG	STD coverage, KM width	ΔSTD depth: −1.98 ± 0.93 mm Complete coverage in 64% of the cases ΔKM width: 2.02 ± 1.05 mm
Yang et al. (2021) [55]	Case report	1	3 y	Digital prosthetic/tunnel flap + SCTG	Buccal STD	Remaining STD of less than 0.5 mm

Abbreviations: m, months; y, years; CAF, coronally advanced flap; CTG, connective tissue graft; STD, soft tissue dehiscence; PPD, pocket probing depth; KM, keratinized mucosa; ADM, acellular dermal matrix; CAL, clinical attachment level; STT, soft tissue thickness; SCTG, subepithelial connective tissue graft; VISTA, vestibular incision supraperiosteal tunnel access; N/A, not appliable; PECTG, partially epithelialized connective tissue graft.

## Data Availability

Not applicable.

## References

[1] Pjetursson B.E., Asgeirsson A.G., Zwahlen M., Sailer I. (2014). Improvements in implant dentistry over the last decade: Comparison of survival and complication rates in older and newer publications. Int. J. Oral Maxillofac. Implant..

[2] Jung R.E., Zembic A., Pjetursson B.E., Zwahlen M., Thoma D.S. (2012). Systematic review of the survival rate and the incidence of biological, technical, and aesthetic complications of single crowns on implants reported in longitudinal studies with a mean follow-up of 5 years. Clin. Oral Implant. Res..

[3] Buser D., Sennerby L., De Bruyn H. (2017). Modern implant dentistry based on osseointegration: 50 years of progress, current trends and open questions. Periodontology 2000.

[4] De Bruyn H., Raes S., Matthys C., Cosyn J. (2015). The current use of patient-centered/reported outcomes in implant dentistry: A systematic review. Clin. Oral Implant. Res..

[5] Belser U.C., Grütter L., Vailati F., Bornstein M.M., Weber H.P., Buser D. (2009). Outcome evaluation of early placed maxillary anterior single-tooth implants using objective esthetic criteria: A cross-sectional, retrospective study in 45 patients with a 2- to 4-year follow-up using pink and white esthetic scores. J. Periodontol..

[6] Fürhauser R., Florescu D., Benesch T., Haas R., Mailath G., Watzek G. (2005). Evaluation of soft tissue around single-tooth implant crowns: The pink esthetic score. Clin. Oral Implant. Res..

[7] Cosyn J., Thoma D.S., Hämmerle C.H., De Bruyn H. (2017). Esthetic assessments in implant dentistry: Objective and subjective criteria for clinicians and patients. Periodontology 2000.

[8] Zucchelli G., Barootchi S. (2021). Implant soft tissue Dehiscence coverage Esthetic Score (IDES): A pilot within- and between-rater analysis of consistency in objective and subjective scores. Clin. Oral Implant. Res..

[9] Roccuzzo M., Gaudioso L., Bunino M., Dalmasso P. (2014). Surgical treatment of buccal soft tissue recessions around single implants: 1-year results from a prospective pilot study. Clin. Oral Implant. Res..

[10] Zucchelli G., Mazzotti C., Mounssif I., Mele M., Stefanini M., Montebugnoli L. (2013). A novel surgical-prosthetic approach for soft tissue dehiscence coverage around single implant. Clin. Oral Implant. Res..

[11] Berglundh T., Armitage G., Araujo M.G., Avila-Ortiz G., Blanco J., Camargo P.M., Chen S., Cochran D., Derks J., Figuero E. (2018). Peri-implant diseases and conditions: Consensus report of workgroup 4 of the 2017 World Workshop on the Classification of Periodontal and Peri-Implant Diseases and Conditions. J. Clin. Periodontol..

[12] Mazzotti C., Stefanini M., Felice P., Bentivogli V., Mounssif I., Zucchelli G. (2018). Soft-tissue dehiscence coverage at peri-implant sites. Periodontology 2000.

[13] Zucchelli G., Tavelli L. (2019). Classification of facial peri-implant soft tissue dehiscence/deficiencies at single implant sites in the esthetic zone. J. Periodontol..

[14] Barootchi S., Mancini L. (2022). Reliability assessment of the classification for facial peri-implant soft tissue dehiscence/deficiencies (PSTDs): A multi-center inter-rater agreement study of different skill-level practitioners. J. Periodontol..

[15] Bengazi F., Wennström J.L., Lekholm U. (1996). Recession of the soft tissue margin at oral implants. A 2-year longitudinal prospective study. Clin. Oral Implant. Res..

[16] Kan J.Y., Rungcharassaeng K., Lozada J.L., Zimmerman G. (2011). Facial gingival tissue stability following immediate placement and provisionalization of maxillary anterior single implants: A 2- to 8-year follow-up. Int. J. Oral Maxillofac. Implant..

[17] Romandini M., Pedrinaci I., Lima C., Soldini M.C., Araoz A., Sanz M. (2021). Prevalence and risk/protective indicators of buccal soft tissue dehiscence around dental implants. J. Clin. Periodontol..

[18] Sanz-Martín I., Regidor E., Navarro J., Sanz-Sánchez I., Sanz M., Ortiz-Vigón A. (2020). Factors associated with the presence of peri-implant buccal soft tissue dehiscences: A case-control study. J. Periodontol..

[19] Tavelli L., Barootchi S. (2021). Prevalence and risk indicators of midfacial peri-implant soft tissue dehiscence at single site in the esthetic zone: A cross-sectional clinical and ultrasonographic study. J. Periodontol..

[20] Garabetyan J., Malet J., Kerner S., Detzen L. (2019). The relationship between dental implant papilla and dental implant mucosa around single-tooth implant in the esthetic area: A retrospective study. Clin. Oral Implant. Res..

[21] Chen S.T., Buser D. (2009). Clinical and esthetic outcomes of implants placed in postextraction sites. Int. J. Oral Maxillofac. Implant..

[22] Evans C.D., Chen S.T. (2008). Esthetic outcomes of immediate implant placements. Clin. Oral Implant. Res..

[23] Nisapakultorn K., Suphanantachat S., Silkosessak O., Rattanamongkolgul S. (2010). Factors affecting soft tissue level around anterior maxillary single-tooth implants. Clin. Oral Implant. Res..

[24] Cosyn J., Sabzevar M.M., De Bruyn H. (2012). Predictors of inter-proximal and midfacial recession following single implant treatment in the anterior maxilla: A multivariate analysis. J. Clin. Periodontol..

[25] Buser D., Martin W., Belser U.C. (2004). Optimizing esthetics for implant restorations in the anterior maxilla: Anatomic and surgical considerations. Int. J. Oral Maxillofac. Implant..

[26] Benic G.I., Mokti M., Chen C.J., Weber H.P., Hämmerle C.H., Gallucci G.O. (2012). Dimensions of buccal bone and mucosa at immediately placed implants after 7 years: A clinical and cone beam computed tomography study. Clin. Oral Implant. Res..

[27] Sanz Martin I., Benic G.I., Hämmerle C.H., Thoma D.S. (2016). Prospective randomized controlled clinical study comparing two dental implant types: Volumetric soft tissue changes at 1 year of loading. Clin. Oral Implant. Res..

[28] Jung R.E., Herzog M., Wolleb K., Ramel C.F., Thoma D.S., Hämmerle C.H. (2017). A randomized controlled clinical trial comparing small buccal dehiscence defects around dental implants treated with guided bone regeneration or left for spontaneous healing. Clin. Oral Implant. Res..

[29] Miyamoto Y., Obama T. (2011). Dental cone beam computed tomography analyses of postoperative labial bone thickness in maxillary anterior implants: Comparing immediate and delayed implant placement. Int. J. Periodontics Restor. Dent..

[30] Roccuzzo M., Grasso G., Dalmasso P. (2016). Keratinized mucosa around implants in partially edentulous posterior mandible: 10-year results of a prospective comparative study. Clin. Oral Implant. Res..

[31] Wennström J.L., Derks J. (2012). Is there a need for keratinized mucosa around implants to maintain health and tissue stability?. Clin. Oral Implant. Res..

[32] Warrer K., Buser D., Lang N.P., Karring T. (1995). Plaque-induced peri-implantitis in the presence or absence of keratinized mucosa. An experimental study in monkeys. Clin. Oral Implant. Res..

[33] Lin G.H., Chan H.L., Wang H.L. (2013). The significance of keratinized mucosa on implant health: A systematic review. J. Periodontol..

[34] Veltri M., Ekestubbe A., Abrahamsson I., Wennström J.L. (2016). Three-Dimensional buccal bone anatomy and aesthetic outcome of single dental implants replacing maxillary incisors. Clin. Oral Implant. Res..

[35] Kan J.Y.K., Rungcharassaeng K., Deflorian M., Weinstein T., Wang H.L., Testori T. (2018). Immediate implant placement and provisionalization of maxillary anterior single implants. Periodontology 2000.

[36] Chen S.T., Buser D. (2014). Esthetic outcomes following immediate and early implant placement in the anterior maxilla—A systematic review. Int. J. Oral Maxillofac. Implant..

[37] De Rouck T., Collys K., Cosyn J. (2008). Immediate single-tooth implants in the anterior maxilla: A 1-year case cohort study on hard and soft tissue response. J. Clin. Periodontol..

[38] Hämmerle C.H., Araújo M.G., Simion M. (2012). Evidence-based knowledge on the biology and treatment of extraction sockets. Clin. Oral Implant. Res..

[39] Raes F., Cosyn J., Crommelinck E., Coessens P., De Bruyn H. (2011). Immediate and conventional single implant treatment in the anterior maxilla: 1-year results of a case series on hard and soft tissue response and aesthetics. J. Clin. Periodontol..

[40] De Rouck T., Collys K., Wyn I., Cosyn J. (2009). Instant provisionalization of immediate single-tooth implants is essential to optimize esthetic treatment outcome. Clin. Oral Implant. Res..

[41] den Hartog L., Raghoebar G.M., Stellingsma K., Vissink A., Meijer H.J. (2011). Immediate non-occlusal loading of single implants in the aesthetic zone: A randomized clinical trial. J. Clin. Periodontol..

[42] Canullo L., Iurlaro G., Iannello G. (2009). Double-blind randomized controlled trial study on post-extraction immediately restored implants using the switching platform concept: Soft tissue response. Preliminary report. Clin. Oral Implant. Res..

[43] Zuiderveld E.G., den Hartog L., Vissink A., Raghoebar G.M., Meijer H.J. (2014). Significance of buccopalatal implant position, biotype, platform switching, and pre-implant bone augmentation on the level of the midbuccal mucosa. Int. J. Prosthodont..

[44] Raes S., Rocci A., Raes F., Cooper L., De Bruyn H., Cosyn J. (2015). A prospective cohort study on the impact of smoking on soft tissue alterations around single implants. Clin. Oral Implant. Res..

[45] Burkhardt R., Joss A., Lang N.P. (2008). Soft tissue dehiscence coverage around endosseous implants: A prospective cohort study. Clin. Oral Implant. Res..

[46] Anderson L.E., Inglehart M.R., El-Kholy K., Eber R., Wang H.L. (2014). Implant associated soft tissue defects in the anterior maxilla: A randomized control trial comparing subepithelial connective tissue graft and acellular dermal matrix allograft. Implant. Dent..

[47] Roccuzzo M., Dalmasso P., Pittoni D., Roccuzzo A. (2019). Treatment of buccal soft tissue dehiscence around single implant: 5-year results from a prospective study. Clin. Oral Investig..

[48] Zucchelli G., Felice P., Mazzotti C., Marzadori M., Mounssif I., Monaco C., Stefanini M. (2018). 5-year outcomes after coverage of soft tissue dehiscence around single implants: A prospective cohort study. Eur. J. Oral Implantol..

[49] Mareque-Bueno S. (2011). A novel surgical procedure for coronally repositioning of the buccal implant mucosa using acellular dermal matrix: A case report. J. Periodontol..

[50] Schallhorn R.A., McClain P.K., Charles A., Clem D., Newman M.G. (2015). Evaluation of a porcine collagen matrix used to augment keratinized tissue and increase soft tissue thickness around existing dental implants. Int. J. Periodontics Restor. Dent..

[51] Cosyn J., De Bruyn H., Cleymaet R. (2013). Soft tissue preservation and pink aesthetics around single immediate implant restorations: A 1-year prospective study. Clin. Implant. Dent. Relat. Res..

[52] Frisch E., Ratka-Krüger P. (2020). A new technique for peri-implant recession treatment: Partially epithelialized connective tissue grafts. Description of the technique and preliminary results of a case series. Clin. Implant. Dent. Relat. Res..

[53] Ueno D., Jayawardena J.A., Kurokawa T. (2015). Peri-implant mucosal dehiscence coverage with a modified semilunar coronary positioned flap in posterior maxilla: A case report. Int. J. Implant. Dent..

[54] Schoenbaum T.R., Solnit G.S., Alawie S., Sadowsky S.J. (2019). Treatment of peri-implant recession with a screw-retained, interim implant restoration: A clinical report. J. Prosthet. Dent..

[55] Yang J., Liu Q., Shiba T., Ji C., Iwata T., Jiang T. (2021). Application of digital prosthodontics and connective tissue grafting in the management of peri-implant mucosal recession around a malpositioned 1-piece implant: A clinical report. J. Prosthet Dent..

[56] Lee C.T., Hamalian T., Schulze-Späte U. (2015). Minimally invasive treatment of soft tissue deficiency around an implant-supported restoration in the esthetic zone: Modified VISTA technique case report. J. Oral Implantol..

[57] Bertl K., Pifl M., Hirtler L., Rendl B., Nürnberger S., Stavropoulos A., Ulm C. (2015). Relative Composition of Fibrous Connective and Fatty/Glandular Tissue in Connective Tissue Grafts Depends on the Harvesting Technique but not the Donor Site of the Hard Palate. J. Periodontol..

[58] Zucchelli G., Mele M., Stefanini M., Mazzotti C., Marzadori M., Montebugnoli L., de Sanctis M. (2010). Patient morbidity and root coverage outcome after subepithelial connective tissue and de-epithelialized grafts: A comparative randomized-controlled clinical trial. J. Clin. Periodontol..

[59] Thoma D.S., Mühlemann S., Jung R.E. (2014). Critical soft-tissue dimensions with dental implants and treatment concepts. Periodontology 2000.

[60] Chackartchi T., Romanos G.E., Sculean A. (2019). Soft tissue-related complications and management around dental implants. Periodontology 2000.

[61] Araújo M.G., Sukekava F., Wennström J.L., Lindhe J. (2005). Ridge alterations following implant placement in fresh extraction sockets: An experimental study in the dog. J. Clin. Periodontol..

[62] Frizzera F., de Freitas R.M., Muñoz-Chávez O.F., Cabral G., Shibli J.A., Marcantonio E. (2019). Impact of Soft Tissue Grafts to Reduce Peri-implant Alterations After Immediate Implant Placement and Provisionalization in Compromised Sockets. Int. J. Periodontics Restor. Dent..

[63] Seyssens L., De Lat L., Cosyn J. (2021). Immediate implant placement with or without connective tissue graft: A systematic review and meta-analysis. J. Clin. Periodontol..

[64] Scribante A., Butera A. (2022). Customized Minimally Invasive Protocols for the Clinical and Microbiological Management of the Oral Microbiota. Microorganisms.

